# AhNPR4B Interacts with AhPR2-Like and May Contribute to Disease Resistance and Cold Tolerance in Peanut

**DOI:** 10.3390/plants15101588

**Published:** 2026-05-21

**Authors:** Xiaoyu Zhang, Xiaoji Zhang, Zhenbo Chen, Rui Zhang, Yunyun Xue, Na Li, Yuexia Tian, Huiqi Zhang, Dongmei Bai, Xin Zhang

**Affiliations:** 1 Shanxi Key Laboratory of Oil Crops Genetic Improvement and Germplasm Enhancement, Institute of Industrial Crops, Shanxi Agricultural University, Taiyuan 030031, China; 20233423@stu.sxau.edu.cn (X.Z.); s20222136@stu.sxau.edu.cn (X.Z.); 202430118@stu.sxau.edu.cn (Z.C.); sxndjzsln@sxau.edu.cn (N.L.); sxndjzstyx@sxau.edu.cn (Y.T.);; 2 College of Plant Protection, Shanxi Agricultural University, Taigu 030801, China; 3 College of Agronomy, Shanxi Agricultural University, Taigu 030801, China

**Keywords:** *Arachis hypogaea* L., *AhNPR4B*, PR protein, disease resistance, cold stress, protein–protein interaction

## Abstract

Peanut (*Arachis hypogaea* L.) production faces persistent threats from various infectious diseases. Planting healthy varieties with robust botanical defense networks is critical for minimizing future costs. Non-expressor of pathogenesis-related (NPR) regulators are involved in immune activation and act as key targets for deeper stress adaptation, and are thus promising targets for genetic enhancement. In this study, we characterized the peanut NPR4B protein and demonstrated its local subcellular binding to the nucleus. Ectopic overexpression of *AhNPR4B* in *Arabidopsis thaliana* significantly enhanced resistance to the necrotrophic pathogen *Botrytis cinerea* and enhanced cold tolerance, as supported by quantitative and statistical analyses (*p* < 0.05). As regards underlying molecular events, Y2H (Yeast 2-Hybrid) analysis revealed a binding in vitro physical relation of AhPR2-like to AhNPR4B. This binding was demonstrated in vivo through BiFC (Bimolecular Fluorescence Complementation). These results suggest that the AhNPR4B-AhPR2-like complex may act as a key regulatory module associated with biotic and abiotic stress signaling, potentially contributing to broad-spectrum stress resistance. These findings provide foundational insights into the functional roles of *AhNPR4B* and its interaction with *AhPR2-like* in regulating stress resistance and support its potential as a candidate target for future genetic improvements to enhance stress resilience in peanuts.

## 1. Introduction

Throughout natural ecosystems, botanical species constantly face diverse biotic and abiotic challenges. Consequently, these detrimental conditions severely restrict fundamental developmental processes, thereby jeopardizing crop yields worldwide, alongside general nutritional security [1]. To survive these fluctuating conditions, plants have evolved highly sophisticated immune mechanisms and stress-signaling networks, allowing them to rapidly sense environmental perturbations and recalibrate their physiological and biochemical states [2]. Non-expressor of pathogenesis-related (NPR) regulators serve as core integration points governing botanical defense system and coordinate key host responses. Such molecular components remain strictly required to propagate signaling cascades reliant on salicylic acid, ultimately triggering systemic acquired resistance, commonly abbreviated as SAR, across the organism [3,4]. In the model plant *Arabidopsis thaliana*, NPR members such as AtNPR3 and AtNPR4 function as salicylic acid (SA) receptors and nuclear transcriptional co-regulators, regulating the transcription of subsequent pathogenesis-related (PR) loci through intermediate or immediate mechanisms, thereby establishing a robust defense against microbial infections [3].

However, as research has progressed beyond simple defense models, cross-regulation of opposing stress responses becomes important in plant biology. It is increasingly believed that plant defense pathways and abiotic stress responses arise not as independent modules but as coordinated or antagonistic partners [5]. Although NPR proteins are well characterized in model species, their role in abiotic stresses, especially cold tolerance, is not widely understood as a mechanistic model [6,7]. Resource allocation demonstrates a fundamental metabolic tradeoff between immunity and physical growth. For example, constitutive activation of SA-NPR1 in rice leads to large yield penalties by suppressing critical growth-related hormones [8].

Peanuts (*Arachis hypogaea* L.), a rich oat oilseed crop of great value, are an even worse problem, with rapid growth times under simultaneous biotic and abiotic pressures [9]. Gray mold is a local infection of the necrotrophic fungus *Botrytis cinerea* that can rapidly kill plants, causing the tissue to freeze and premature flower/pod drop [10]. Groundnuts grown in warm climates are extremely sensitive to cold conditions, and cold snaps in early spring or fall can cause membrane lipid phase changes, excess reactive oxygen species (ROS), sudden degradation of photosynthetic efficiency, and consequently, large yield losses [11].

Traditionally, resistance to necrotrophic pathogens is thought to be governed by jasmonic acid (JA) and ethylene (ET) signaling, which typically act antagonistically to the SA pathway [12]. Nevertheless, emerging multi-omics evidence suggests that the SA-JA antagonism is not an absolute dichotomy [13,14]. Different NPR family members exhibit varying affinities for SA; however, functional divergence is common among NPR4 orthologs in different plant species, and several NPR4 homologs have been reported to act as positive regulators of stress resistance rather than repressors. In *Arabidopsis*, *AtNPR4* has been characterized as a high-affinity salicylic acid receptor that represses defense gene expression under low-SA conditions to balance growth and immunity [3,12]. However, orthologous NPR4 genes from different plant species often exhibit functional diversification, and several NPR4 homologs have been reported to positively regulate stress resistance in a context-dependent manner. As a homolog of *AtNPR4*, which acts as a transcriptional repressor, AhNPR4B may activate stress responses when overexpressed through several potential mechanisms: (1) titration of endogenous repressive proteins, (2) altered stoichiometry of nuclear complexes, and (3) functional divergence during peanut evolution. These possibilities are discussed further in the Discussion section. This suggests that specific NPR orthologs may possess the capacity to break the rigid antagonism between SA and JA through precise hormonal crosstalk, thereby exerting unconventional protective effects against necrotrophs. Concurrently, the intersection of abiotic stress networks and plant immunity has gained traction; low temperatures induce transient increases in endogenous SA, and moderate SA accumulation appears to be essential for activating the C-repeat binding factor (CBF) response cascade [15,16]. Proteins within the PR-2 classification, specifically β-1,3-glucanases, execute biphasic protective strategies. Although they physically dismantle the fungal structural envelopes to directly restrict microbial growth, enzymatic cleavage simultaneously yields short carbohydrate derivatives. These released molecules subsequently operate as damage-associated molecular patterns (DAMPs), thereby intensifying downstream defense pathways [17,18].

However, the roles of peanut NPR genes in these interdependent stress pathways are poorly understood. In the present study, we focused on *AhNPR4B* (an ortholog of *Arabidopsis AtNPR4*) to illustrate its role in disease resistance and cold tolerance. We found that *AhNPR4B* acts as a positive regulator rather than a canonical repressor, enhancing both disease resistance and cold tolerance when overexpressed, likely because of the functional divergence and specific interactions with *AhPR2-like* in peanuts. Intracellular mapping revealed strong AhNPR4B nucleicity, which was consistent with the expected role of AhPR2-like gene expression. Phenotypic characterization of *AhNPR4B*-overexpressing Arabidopsis lines showed that this gene not only enhanced resistance to *B. cinerea* but also mitigated leaf wilting and chlorosis under cold stress, significantly enhancing cold tolerance. Experiments using Y2H and BiFC firmly established the physical binding between AhPR2-like and AhNPR4B, which we tested in intact biological systems and isolated environments. Ultimately, this regulatory component is a driver of widespread protective properties, suggesting that this locus is particularly suitable for engineering highly robust groundnut crops through genetic innovation.

## 2. Results

### 2.1. Subcellular Localization of AhNPR4B to Nucleus

**Figure 1:**
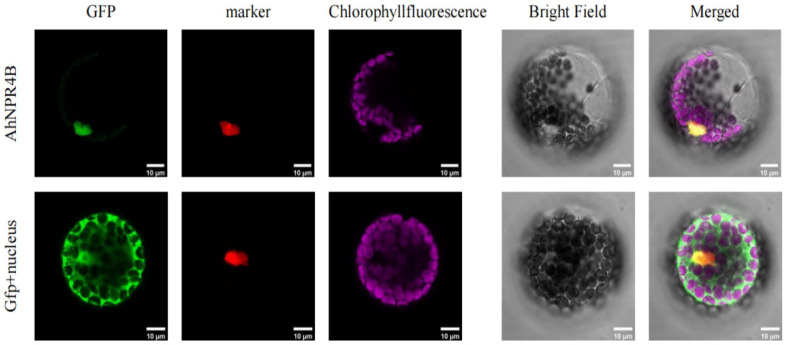
Intracellular distribution profile of the AhNPR4B protein within *Arabidopsis thaliana* protoplasts. An AhNPR4B-GFP fusion expression vector was co-introduced with a nucleus-specific marker into A. thaliana protoplasts via PEG-mediated transfection. Fluorescence signals were observed using confocal laser scanning microscopy. More than 95% of positively transformed cells (*n* > 100) showed exclusive nuclear localization of AhNPR4B-GFP in three independent experiments. Scale bar: 10 μm.

Intracellular spatial profiling involved the transfection of isolated *Arabidopsis thaliana* cells with a recombinant 35S::*AhNPR4B*-GFP vector, followed by short-term evaluation utilizing focal laser scanning microscopy (CLSM). Microscopic observation revealed a green emission pattern strictly isolated within the nucleoplasm. Nuclear localization was further confirmed by colocalization with a nuclear marker. The target-associated signal flawlessly matched the red fluorescence indicator, providing unequivocal proof of absolute nuclear confinement (Figure 1). Chlorophyll autofluorescence was restricted to chloroplasts and did not overlap with the *AhNPR4B*-GFP signal, thereby excluding plastid localization. In contrast, the control group expressing free GFP showed ubiquitous distribution throughout the cell, consistent with its non-targeted nature. The merged images further corroborated the strict nuclear partitioning of *AhNPR4B*, suggesting that it functions as a regulatory protein involved in signal transduction and transcriptional modulation during stress responses. Quantitative analysis showed that exclusive nuclear fluorescence was observed in more than 95% of the transformed cells (*n* > 100 cells observed in three independent experiments), confirming the constitutive nuclear localization of AhNPR4B.

### 2.2. Ectopic Overexpression of AhNPR4B Enhances Resistance to B. cinerea in Arabidopsis

To investigate the immunological functions of AhNPR4B, two independent homozygous T_3_ lines (OE2 and OE3) with stable inheritance and consistent expression levels were selected for phenotypic analysis. In *Arabidopsis*, two independent transgenic lines with consistent phenotypes are generally considered sufficient to validate gene function. Seven-day-old seedlings were inoculated with a *Botrytis cinerea* spore suspension and maintained under high humidity, whereas control plants were mock-inoculated with sterile water.

**Figure 2:**
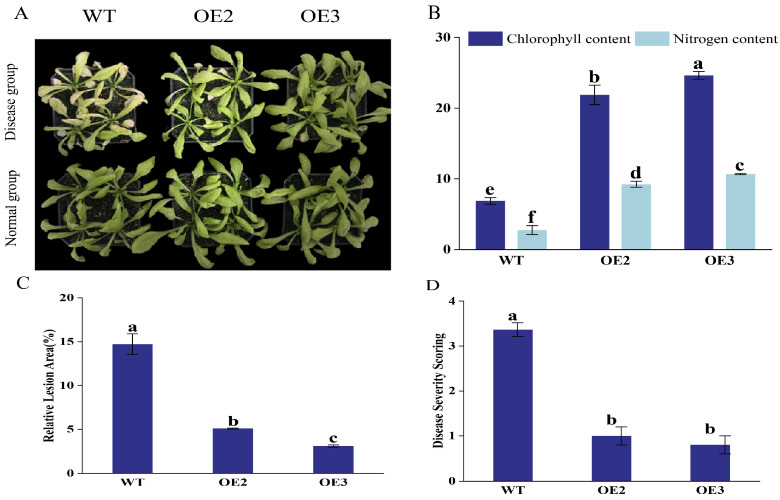
Phenotypic and physiological responses of wild-type (WT) and AhNPR4B-overexpressing (OE2, OE3) *Arabidopsis thaliana* plants to *Botrytis cinerea* infection. (**A**) Representative photographs of WT and transgenic plants under disease and normal conditions. Scale bar = 1 cm. To investigate the immunological function of AhNPR4B, two independent homozygous T3 lines (OE2 and OE3) with stable inheritance and consistent expression levels were selected for phenotypic analysis. In *Arabidopsis*, two independent transgenic lines with consistent phenotypes are generally considered sufficient to validate gene function. Seven-day-old seedlings were inoculated with a *B. cinerea* spore suspension and maintained under high humidity, while control plants were mock-inoculated with sterile water. Under disease pressure, WT plants exhibited severe symptoms, including leaf curling, chlorosis, and wilting, whereas OE2 and OE3 lines showed markedly enhanced resistance, with milder or no symptoms. (**B**–**D**) Quantification of chlorophyll content, nitrogen content, relative lesion area, and disease severity scoring in WT and transgenic plants after inoculation. Data are presented as means ± standard error (SE) from three independent biological replicates. Different lowercase letters indicate significant differences (*p* < 0.05, one-way ANOVA followed by Tukey’s HSD test). Representative images are shown; consistent results were obtained in all replicates.

Under disease pressure, wild-type (WT) plants exhibited severe symptoms, including leaf curling, chlorosis, and wilting, whereas AhNPR4B-overexpressing lines (OE2 and OE3) showed markedly enhanced resistance, with mild or no symptoms (Figure 2A). Consistent with the visual phenotypes, physiological and molecular quantification confirmed the protective effects of AhNPR4B. Chlorophyll and nitrogen contents, which reflect plant vitality under stress, were significantly higher in OE2 and OE3 plants than in WT plants after inoculation (Figure 2B). The relative lesion area was significantly reduced in the transgenic lines (Figure 2C), and disease severity scoring confirmed that WT plants suffered more severe infections than the overexpression lines (Figure 2D). Collectively, these results indicate that the ectopic overexpression of AhNPR4B significantly enhances plant resistance to the necrotrophic fungus *B. cinerea*.

### 2.3. AhNPR4B Overexpression Confers Enhanced Cold Tolerance

**Figure 3:**
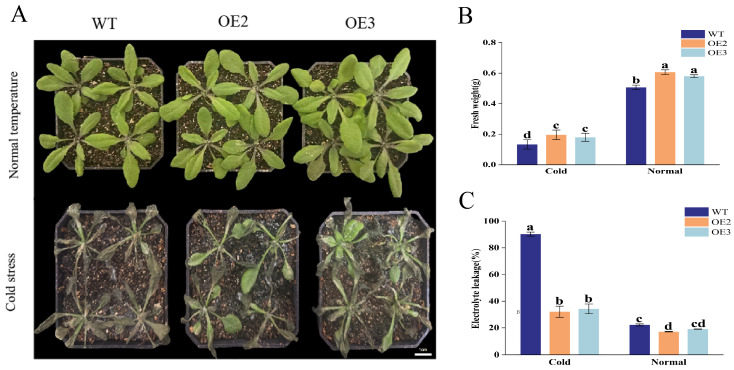
Phenotypic and physiological responses of wild-type (WT) and AhNPR4B-overexpressing (OE2, OE3) *Arabidopsis thaliana* plants under cold stress. (**A**) Representative photographs of plants grown under normal temperature conditions (upper panel) and after cold stress treatment (lower panel). Scale bar = 1 cm. Under cold stress, WT plants exhibited severe wilting, necrosis, and leaf damage, whereas OE2 and OE3 lines retained more green tissue and showed enhanced chilling tolerance. (**B**,**C**) Quantification of fresh weight and electrolyte leakage in WT and transgenic plants under normal and cold conditions. Data are presented as means ± standard error (SE) from three independent biological replicates. Different lowercase letters indicate significant differences (*p* < 0.05, one-way ANOVA followed by Tukey’s HSD test). Representative images are shown; consistent results were obtained in all replicates.

Based on the known roles of NPR family regulators in plant defense and the crosstalk between biotic and abiotic stress responses, we investigated the potential function of AhNPR4B in adaptation to low-temperature stress (Figure 3). Two-week-old seedlings of wild-type (WT) and AhNPR4B-overexpressing lines (OE2, OE3) were subjected to 4 °C cold stress for 7 consecutive days. Under cold conditions, the WT plants exhibited severe wilting, necrosis, and chlorosis, accompanied by significant growth inhibition. In contrast, the transgenic lines showed markedly improved cold tolerance; OE2 plants displayed reduced wilting compared to the WT, whereas OE3 plants retained healthier green leaves and showed higher resilience to chilling injury (Figure 3A).

To quantify these phenotypic differences, we measured the physiological indicators related to cold tolerance. Under cold stress, the fresh weight of both OE2 and OE3 plants was significantly higher than that of the WT plants (Figure 3B), indicating better growth performance and biomass retention. Electrolyte leakage, an indicator of cell membrane damage under stress, was significantly lower in the transgenic lines than in the WT plants after cold treatment (Figure 3C). These physiological data further supported the visual observation that AhNPR4B overexpression alleviated cold-induced damage.

Collectively, these results demonstrated that AhNPR4B functions as a versatile molecular regulator that enhances both disease resistance and cold tolerance, suggesting its potential role in coordinating signal transduction networks for both environmental resilience and pathogen defense.

### 2.4. Identification of the In Vitro Interaction Between AhNPR4B and AhPR2-Like via Yeast Two-Hybrid (Y2H) Assays

**Figure 4:**
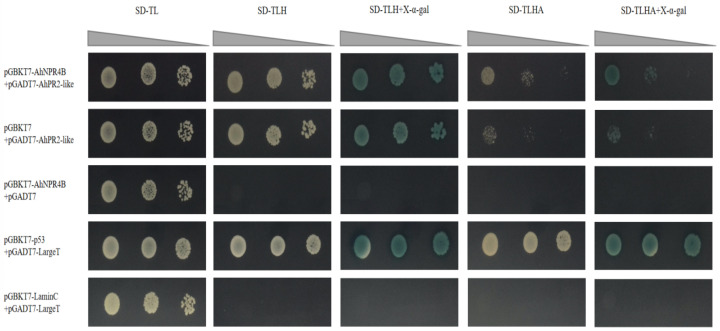
Direct molecular coupling linking AhPR2-like with AhNPR4B demonstrated via Y2H methodology. Colony proliferation profiles among dual-vector carrying strains were monitored utilizing gradient nutrient-restrictive agars. Specifically, baseline plasmid internalization was established by culturing on double-dropout platforms (SD-TL, lacking Trp and Leu) to confirm successful co-transformation. The actual interaction was evaluated based on the growth and blue colony formation on SD-TLH (lacking Trp, Leu, and His) and SD-TLHA (lacking Trp, Leu, His, and Ade) agar plates supplemented with 0.25 mM 3-AT and/or X-α-gal.

Decoding the interaction networks governed by AhNPR4B necessitated the systematic interrogation of an Arachis hypogaea complementary DNA archive using Y2H screening protocols. We identified a pathogenesis-related protein (NCBI Gene ID: LOC112723217), hereafter designated AhPR2-like, as a potential interacting partner. Targeted pairwise yeast evaluation verified these physical associations across a gradient of nutritional selection pressures. Baseline viability was initially established by vigorous colony expansion on double-dropout agar (SD-TL) across all evaluated lineages. This cohort included the primary pGBKT7-AhNPR4B/pGADT7-AhPR2-like combination, recognized p53/LargeT validation pairs, and empty vector backgrounds. Under subsequent severely restrictive screening, the specific AhNPR4B/AhPR2-like transformants and recognized positive indicators maintained survival. Subsequent plating on severely restricted nutritional platforms (SD-TLHA) revealed sustained proliferation exclusively among the target co-transformants, along with recognized validation pairings. These specific colonies simultaneously displayed distinct chromogenic conversion following X-α-gal supplementation, signifying robust molecular coupling. As expected, background references utilizing empty vector constructs demonstrated zero viability across rigorous selection criteria (Figure 4). These analytical readouts provide definitive biochemical validation of the physical attachment of AhPR2-like to AhNPR4B.

### 2.5. In Vivo Interaction Analysis of AhNPR4B and AhPR2-Like via Bimolecular Fluorescence Complementation (BiFC) Assays

**Figure 5:**
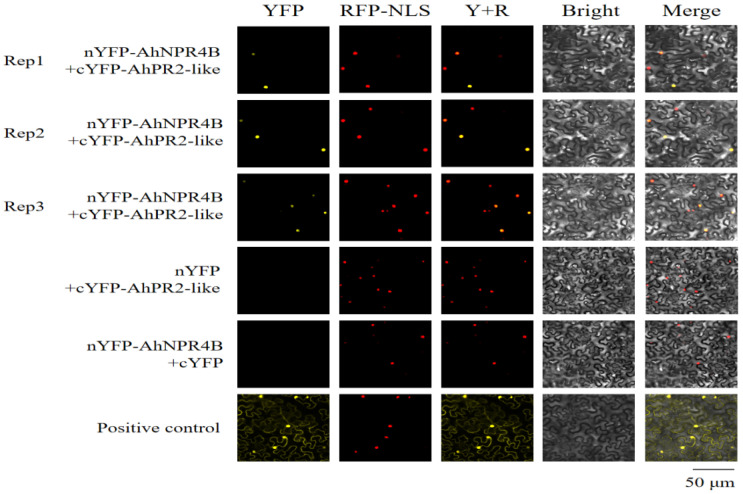
Intracellular validation demonstrating direct physical coupling linking AhPR2-like with AhNPR4B via BiFC analysis. Designated recombinant plasmids underwent simultaneous transient delivery into the epidermal tissue of *Nicotiana benthamiana* foliage. Yellow fluorescent signals indicate successful protein interactions, while the red fluorescent signal from the RFP-NLS construct indicates the location of the cell nucleus. The observation was consistent across three independent biological replicates (Rep1 to Rep3). Combinations of nYFP + cYFP-AhPR2-like and nYFP-AhNPR4B + empty cYFP served as negative background controls, whereas a known interacting pair was used as the positive control. Scale bar = 50 μm. Three independent biological replicates were performed, and consistent nuclear fluorescence signals were detected in all replicates.

For in planta verification of this physical binding, BiFC techniques were implemented using transient agroinfiltration across Nicotiana benthamiana foliage. Subsequent microscopic evaluation revealed robust yellow emission patterns strictly confined to the nucleoplasm upon simultaneous introduction of cYFP-AhPR2-like and nYFP-AhNPR4B. Such targeted spatial signaling directly corresponds to the recognized intranuclear residency of this specific regulatory factor (Figure 5). No significant fluorescence was detected in the negative control combinations (nYFP-AhNPR4B + cYFP or nYFP + cYFP-AhPR2-like), confirming the specificity of the interaction and validity of the experimental system. These results support a physical interaction between AhPR2-like and *AhNPR4B*. However, genetic evidence directly linking this interaction to the observed phenotypes is lacking, and the role of *AhPR2-like* in stress tolerance remains unclear. Thus, we do not claim a definitive causal role but propose that this interaction may underpin the enhanced stress tolerance observed in this study. Overexpression of *AhNPR4B* alone may cause phenotypes unrelated to the native complex, which should be noted.

## 3. Discussion

Throughout their natural life cycle, plants are inevitably subjected to the combined effects of fluctuating climate and pathogenic microorganisms. A central challenge in plant molecular biology is understanding how plants optimize resource allocation to trigger broad-spectrum resistance against both biotic and abiotic stresses within complex environments [19]. In this study, systematic identification of the peanut *AhNPR4B* gene not only revealed its critical pleiotropic role in defending against the necrotrophic fungus *Botrytis cinerea* and enhancing cold stress tolerance, but also demonstrated, for the first time, a direct physical interaction between AhNPR4B and an AhPR2-like protein within the nucleus. This finding provides a novel theoretical perspective for re-evaluating cross-stress signaling networks mediated by the NPR family.

In the canonical plant immune signaling pathway, the activation of salicylic acid (SA) signaling relies heavily on dynamic changes in the subcellular localization of NPR proteins. In the model plant *Arabidopsis thaliana*, for instance, *AtNPR1* predominantly resides in the cytoplasm as an oligomer under resting conditions. Triggered by salicylic acid bursts during microbial invasion, these multimeric protein complexes structurally disassemble into independent subunits. Following targeted import across the nuclear envelope, these singular forms physically engage TGA-family regulatory elements, an association that effectively stimulates the promoter activation of subsequent pathogenesis-related targets [20]. However, orthologous NPR4 genes frequently exhibit functional divergence among plant species. Unlike *AtNPR4*, *AhNPR4B* acts as a positive regulator of disease resistance and cold tolerance, which may be attributed to evolutionary adaptation and specific interactions with *AhPR2-like* proteins in peanuts. Unlike *AtNPR4*, which acts as a transcriptional repressor under low-SA conditions, *AhNPR4B* positively regulates disease resistance and cold tolerance. As a homolog of a repressor-type NPR, *AhNPR4B* may switch to an activator when overexpressed through several possible mechanisms, including the titration of endogenous repressive components, altered stoichiometry of nuclear complexes, and evolutionary neofunctionalization in peanuts.

Notably, our results showed that the *AhNPR4B*-GFP fusion protein exhibited strict nuclear-specific localization with no detectable cytoplasmic fluorescence. This strong constitutive nuclear localization suggests that *AhNPR4B* may not follow the nucleocytoplasmic shuttling regulatory model of *NPR1* but rather functions predominantly within the nuclear compartment. Based on its functional domains, we hypothesized that nuclear-localized *AhNPR4B* is involved in chromatin remodeling or assembly of transcriptional complexes. Following encounters with microbial virulence factors or acute chilling episodes, we hypothesized that *AhNPR4B* undergoes rapid post-translational modifications, such as phosphorylation or ubiquitination, to potentially switch its role from a repressor to an activator, thereby facilitating instantaneous responses to stress signals [21]. This sustained nuclear presence provides the spatial rationality required for the protein to simultaneously intervene in both the rapid physical changes induced by the environment and biological invasions.

To investigate macroscopic protective capabilities, engineered *Arabidopsis* backgrounds featuring amplified *AhNPR4B* transcription were subjected to targeted inoculation with *B. cinerea*. This pathological assay verified the functional and biological significance of the aforementioned molecular coupling across intact host organisms. *B. cinerea* is a typical broad-host-range necrotrophic fungus, and it is generally accepted that resistance to such fungi depends on synergistic JA/ET signaling pathways, whereas overactivation of the SA pathway often leads to increased susceptibility due to hormonal antagonism [12]. Interestingly, our phenotypic characterization revealed that *AhNPR4B*-overexpressing lines exhibited significantly smaller lesions and a marked reduction in leaf curling and chlorosis. When plants face simultaneous or sequential stress, these regulators can fine-tune the spatiotemporal distribution of SA and JA concentrations by modulating their transcriptional activity [22,23]. We propose that *AhNPR4B* overexpression does not induce a blind or excessive SA burst; instead, it establishes a more sensitive basal “priming” state. Within this specific physiological context, upon host perception of structurally degrading proteins alongside phytotoxins exuded from *B. cinerea*, the host can rapidly mobilize the localized expression of direct hydrolases, such as AhPR2-like. We hypothesized that the AhNPR4B-AhPR2-like complex in the nucleus could silence genes that inhibit key defense pathways, which might indirectly release constraints on necrotrophic fungal defense signals [24]. This explains why the overexpressing plants did not exhibit the typical trade-offs associated with strong resistance genes, such as dwarfism or delayed development, but instead achieved a more balanced broad-spectrum tolerance [22,25].

Similar to its immunity and in the case of *AhNPR4B*-overexpressing plants, we tested the cold tolerance of *AhNPR4B*. Cold stress often causes endoplasmic reticulum stress and reactive oxygen species (ROS) bursts in plants, with SA being a molecule that regulates endogenous redox homeostasis [26,27,28]. Consistent with its nuclear localization, AhNPR4B may integrate immune and cold stress signaling by interacting with AhPR2-like in the nucleus. These observations supported the hypothesis that AhNPR4B acts as a molecular hub connecting biotic and abiotic stress responses. The detailed regulatory mechanisms and downstream pathways need to be explored in future studies. These results indicated that *AhNPR4B* serves not only as a defense force for the immune response but also as a link between temperature-sensing networks and disease control [29,30].

Central to our current findings is the definitive structural coupling linking the AhPR2-like and AhNPR4B proteins. Intracellular evaluations via BiFC techniques, complemented by Y2H screening, firmly established this physical attachment while verifying its exclusive occurrence inside intranuclear environments. Traditional plant pathology posits that PR2-family proteins are primarily secreted into the apoplast or stored in vacuoles to exert direct antimicrobial effects by degrading the β-1,3-glucan backbone of fungal cell walls [17]. The interaction of a secretory pathway protein with a core transcriptional co-regulator in the nucleus challenges the conventional unidirectional regulatory paradigm. Recent advances in nuclear proteomics have revealed that an increasing number of cytoplasmic and secretory enzymes have been shown to possess moonlighting functions, executing inward relocation across the nuclear envelope to govern subsequent transcriptional networks [31]. For example, certain PR defense proteins have been observed to accumulate in the nucleus upon external stimuli [32]. Although this hypothesis requires further validation by immunoprecipitation and protein degradation assays, it provides a compelling new example of subcellular functional diversification [33]. In this study, we focused on the interaction between AhNPR4B and AhPR2-like and its role in stress tolerance. The independent functional characterization of AhPR2-like, including its subcellular localization in peanuts, overexpression phenotype, and detailed expression patterns under biotic and abiotic stresses, will be systematically explored in our future work.

A limitation of the present study is that all functional characterizations were performed in the heterologous *Arabidopsis thaliana* system without corresponding genetic or expression validation in the native host peanut. Although ectopic expression in *Arabidopsis* provides clear evidence for the conserved roles of *AhNPR4B* in disease resistance and cold tolerance, future studies are required to directly characterize its expression patterns, regulatory mechanisms, and biotechnological potential in peanuts under relevant field and laboratory conditions, as well as to determine the expression pattern of *AhPR2*-like in response to *Botrytis cinerea* infection and cold stress.

Due to the low genetic transformation efficiency and long growth cycle of peanuts, loss-of-function analyses, such as CRISPR/Cas9-mediated knockout and complementary rescue experiments, were not completed in the current study. In future studies, we plan to verify the endogenous function of AhNPR4B in peanuts using gene editing and complementary lines.

In the present study, we focused on identifying the identification of *AhNPR4B* function and its interaction with AhPR2-like proteins. Independent functional characterization of AhPR2-like, including its subcellular localization in peanuts, overexpression phenotype, and stress-induced expression patterns, will be systematically investigated in future studies.

To further verify the functional relevance of the AhNPR4B–AhPR2-like interaction, additional experiments will be performed in future studies: silencing or knockout of *AhPR2-like* in *AhNPR4B*-overexpression lines to determine whether the enhanced stress tolerance depends on AhPR2-like, co-immunoprecipitation (Co-IP) assays to detect the interaction under pathogen infection and cold stress conditions, and genetic rescue or complementation assays to confirm the in vivo function of the AhNPR4B–AhPR2-like complex.

In summary, although our results provide reliable experimental evidence for the model plant Arabidopsis and other interaction assays, the full molecular regulatory network of AhNPR4B remains unclear. Future studies based on combined transcriptomic and metabolomic analyses of peanut overexpression and knockout lines are required to identify the exact downstream target sequences of this multi-stress crosstalk hub to enable molecular design breeding for crop resiliency.

## 4. Materials and Methods

### 4.1. Subcellular Localization and Colocalization

The full-length coding sequences of AhNPR4B (Phytozome Gene ID: Arahy.KS67M3, 2791 bp) and AhPR2-like (NCBI Gene ID: LOC112723217, 1254 bp) were amplified from peanut cDNA. The full-length CDS of AhNPR4B without a stop codon was cloned into the pCAMBIA1300-GFP vector to construct a 35S::AhNPR4B-GFP fusion vector. The recombinant plasmid was then transferred into Arabidopsis protoplasts via PEG-mediated transfection. Fluorescence signals were observed using a confocal laser-scanning microscope (Leica TCS SP8 Leica Microsystems, Wetzlar, Germany). The GFP fluorescence was detected after excitation at 488 nm. The nuclear marker mCherry-NLS was used for co-localization [34,35]. The expression of the fusion protein was indicated by GFP fluorescence signals; however, Western blot validation was not performed.

### 4.2. Generation of Transgenic Plants

Wild-type Col-0 Arabidopsis plants were genetically modified by standard floral dipping. Cucumber necrosis virus infection was carried out with *Agrobacterium tumefaciens* (specifically, the GV3101 lineage), which was dipped well in our 35S::*AhNPR4B*-GFP vector [36,37]. T0 seeds were screened on 1/2 MS medium, and T_1_ seedlings were transplanted into soil (nutrient soil: vermiculite, 1:1). Transgenic lines were selected on Murashige and Skoog (MS) medium containing 50 mg/L hygromycin. T_2_ lines with a 3:1 segregation ratio were identified, and homozygous T_3_ lines were identified using RT-qPCR and used for phenotypic analyses.

### 4.3. Plant Materials and Growth Conditions

Wild-type *Arabidopsis thaliana* ecotype Col-0 was used as the background for transgenic plant generation. Transgenic lines were generated using Agrobacterium-mediated floral dip transformation. Transgenic seeds were screened on MS medium containing 50 mg/L hygromycin, and homozygous T_3_ lines were obtained through continuous selection of antibiotic-resistant progenies for three generations. Prior to planting in fertilized pots, dry seeds were laid during overnight temperatures (4 °C) in closed-environment incubators (22–24 °C). These incubators provided long-day photoperiods (16 h of full light vs. 8 h of full light) and photosynthetic photon flux densities (120 μmol·m^−2^·s^−1^) [38]. Parallel growth of Nicotiana benthamiana used different physiological conditions: 14 h per day (22–25 °C) and 10 h per night (18–20 °C). Such foliage could be used for short-term agroinfiltration when the plants reach developmental maturity at approximately four or six weeks, usually with five full-spanned leaves [39].

### 4.4. Functional Analysis of Arabidopsis AhNPRs Under Pathogen Stress

*Botrytis cinerea* was recovered from stock cultures stored at −80 °C, activated on potato dextrose agar (PDA) medium, and cultured at 22 °C to induce sufficient sporulation. Fungal spores were collected and filtered through sterile cheesecloth, then adjusted to a final concentration of 1.0 × 10^6^ spores/mL for inoculation [40]. In this study, 7-day-old Arabidopsis seedlings were used for the preliminary *Botrytis* infection tests to ensure uniform leaf expansion, whereas 10-day-old seedlings were used for the main *Botrytis* infection trials because this stage provided sufficient biomass to ensure stable and reproducible inoculation. Seedlings were used in the preliminary tests to verify uniform leaf expansion. The spore suspension was evenly sprayed onto the aerial tissues of wild-type (WT) and *AhNPR4B* overexpression (OE) lines. The inoculated plants were kept in the dark for 24 h to maintain high humidity and then returned to standard growth conditions. Disease phenotypes were evaluated at 7 days post inoculation, and disease symptoms were evaluated by disease severity score, lesion area measurement, chlorophyll content, and nitrogen content. All experiments were performed with three independent biological replicates, and at least 20 plants per genotype were analyzed in each replicate [41]. Disease severity scores were rated on a scale of 0–4 according to the percentage of lesion area: 0 = no visible symptoms; 1 = ≤10% leaf area with lesions or chlorosis; 2 = 11–25% leaf area with lesions or chlorosis; 3 = 26–50% leaf area with lesions or chlorosis; 4 = ≥51% leaf area with lesions, severe wilting or necrosis. Lesion area was measured using ImageJ v1.53t (National Institutes of Health, Bethesda, MD, USA) and calculated as the percentage of lesion area relative to total leaf area. Chlorophyll and nitrogen contents of the infected plants were measured using a chlorophyll meter.

### 4.5. Functional Analysis of Arabidopsis AhNPRs Under Cold Stress

Uniform two-week-old Arabidopsis seedlings grown under standard conditions were subjected to cold stress at 4 °C for 7 consecutive days. No recovery period was included, and phenotypic observations and scoring were conducted immediately after the 7-day cold treatment period. Cold tolerance was evaluated by measuring fresh weight and relative electrolyte leakage, with at least 20 plants per genotype in each replicate. All experiments were performed with at least three independent biological replicates, with *n* ≥ 20 plants per genotype in each replicate, and consistent growth conditions were maintained before stress treatment [42,43]. Fresh weight was determined using an analytical balance after harvesting the whole seedlings. Relative electrolyte leakage was measured using a conductivity meter; the initial conductivity (EC1) was recorded after soaking the leaves in deionized water for 2 h, and the final conductivity (EC2) was recorded after boiling for 15 min. Relative electrolyte leakage (%) = (EC1/EC2) × 100.

### 4.6. Yeast Two-Hybrid (Y2H) Assay

The CDS of AhNPR4B was cloned into the pGBKT7 (DNA-binding domain, BD Biosciences) vector as bait. The CDS of AhPR2-like was cloned into the pGADT7 (activation domain) vector as prey. Recombinant plasmids were co-transformed into yeast strain AH109 using the lithium acetate method [44]. Yeast cells were cultured on SD/-Trp/-Leu (double dropout) medium for transformation selection, and on SD/-Trp/-Leu/-His/-Ade (quadruple dropout) medium with or without X-α-gal for interaction screening. Yeast cells were incubated at 30 °C for 4 days [45].

### 4.7. Bimolecular Fluorescence Complementation (BiFC) Assay

Complete open reading frames encoding AhNPR4B alongside AhPR2-like frames underwent directed insertion, generating precise fusions within the pCAMBIA1300-nYFP and the corresponding pCAMBIA1300-cYFP vector backbones. Following successful incorporation into the *A. tumefaciens* GV3101 lineage, these recombinant cultures were mixed for simultaneous agroinfiltration, targeting the foliage of four-week-old *N. benthamiana* specimens. After 48 h of culture at 24 °C post-delivery, reconstituted yellow emission signals localized within the lower epidermal layer were systematically documented using an Olympus BX43 (Olympus Corporation, Tokyo, Japan) confocal laser-scanning imaging system [46,47].

### 4.8. Statistical Analysis

All quantitative data were processed using Microsoft Excel 2019 and statistically analyzed using IBM SPSS Statistics software (version 26.0). One-way analysis of variance (ANOVA) was performed, followed by Tukey’s honest significant difference (HSD) test for multiple comparisons. Data are presented as means ± standard error (SE) of three independent biological replicates. Differences were considered statistically significant at *p* < 0.05.

## 5. Conclusions

In summary, our results reveal new functional interactions between *AhNPR4B* and *AhPR2-like* activity in the nucleus and further position *AhNPR4B* as a key molecular link connecting these two adaptive networks required to cope with biological and physical threats. Revealing the crosstalk between immunity and cold tolerance offers strong experimental proof of the concept of multi-stress signaling plasticity in plants. Finally, AhNPR4B is a promising field for molecular design breeding as a pathway for developing resilient high-performance crop varieties that thrive under challenging conditions.

## Data Availability

Data will be made available on request.
